# The Brain Effects of Laser Acupuncture in Healthy Individuals: An fMRI Investigation

**DOI:** 10.1371/journal.pone.0012619

**Published:** 2010-09-07

**Authors:** Im Quah-Smith, Perminder S. Sachdev, Wei Wen, Xiaohua Chen, Mark A. Williams

**Affiliations:** 1 School of Psychiatry, Faculty of Medicine, University of New South Wales, Randwick, New South Wales, Australia; 2 Neuropsychiatric Institute, Prince of Wales Hospital, Randwick, New South Wales, Australia; 3 Brain & Ageing Research Program, School of Psychiatry, University of New South Wales, Randwick, New South Wales, Australia; 4 Macquarie Centre for Cognitive Sciences, Macquarie University, Sydney, New South Wales, Australia; Cuban Neuroscience Center, Cuba

## Abstract

**Background:**

As laser acupuncture is being increasingly used to treat mental disorders, we sought to determine whether it has a biologically plausible effect by using functional magnetic resonance imaging (fMRI) to investigate the cerebral activation patterns from laser stimulation of relevant acupoints.

**Methodology/Principal Findings:**

Ten healthy subjects were randomly stimulated with a fibreoptic infrared laser on 4 acupoints (LR14, CV14, LR8 and HT7) used for depression following the principles of Traditional Chinese Medicine (TCM), and 1 control non-acupoint (sham point) in a blocked design (alternating verum laser and placebo laser/rest blocks), while the blood oxygenation level-dependent (BOLD) fMRI response was recorded from the whole brain on a 3T scanner. Many of the acupoint laser stimulation conditions resulted in different patterns of neural activity. Regions with significantly increased activation included the limbic cortex (cingulate) and the frontal lobe (middle and superior frontal gyrus). Laser acupuncture tended to be associated with ipsilateral brain activation and contralateral deactivation that therefore cannot be simply attributed to somatosensory stimulation.

**Conclusions/Significance:**

We found that laser stimulation of acupoints lead to activation of frontal-limbic-striatal brain regions, with the pattern of neural activity somewhat different for each acupuncture point. This is the first study to investigate laser acupuncture on a group of acupoints useful in the management of depression. Differing activity patterns depending on the acupoint site were demonstrated, suggesting that neurological effects vary with the site of stimulation. The mechanisms of activation and deactivation and their effects on depression warrant further investigation.

## Introduction

Despite the remarkable developments in Western Medicine in modern times, public interest in Traditional, Complementary and Alternative Medicine (TCAM), such as acupuncture, remains high [1], [2]. This may be because TCAM is perceived as holistic and relatively free of adverse effects. However, these treatments sit uncomfortably alongside scientific medicine because of strikingly different explanatory systems and the empirical tests applied by each discipline. In order to bridge the gulf between high public acceptability and the lack of empirical evidence for many of these treatments, it is important to reconcile them with modern scientific concepts. Our focus here is on laser acupuncture, and we address the question whether laser acupuncture produces brain effects that are biologically plausible.

There have been many studies [3]–[20] some of which have involved functional magnetic resonance imaging (fMRI) and positron emission tomography (PET) of the brain effects of needle acupuncture. Some neuroimaging and neuroendocrine studies have suggested that needle acupuncture affects hypothalamic as well as extrahypothalamic functions and modulates mood [3], [5]–[9]. Needling of the leg acupoint ST36 and the hand acupoint LI4 was shown to activate the hypothalamus and nucleus accumbens and deactivate the rostral anterior cingulate cortex (rACC) [3]. Superficial needling (i.e. needling that did not produce the classical *de qi* sensation - tingling, numbness or other sensations that occur after an acupuncture needle has been properly placed in the body) and non-acupoints (i.e. points on the skin that do not lie on recognized meridians in Traditional Chinese Medicine [TCM]) did not activate the hypothalamus [7]. Stimulation of the acupoint PC6, located above the wrist, recommended for a wide range of conditions from nausea to stress management, resulted in activation of the cerebellum, dorsomedial nucleus of the thalamus, anterior cingulate gyrus and left superior frontal gyrus [8]. All of these studies have used needle acupuncture which, although more traditional, is invasive.

While laser acupuncture has become an increasingly common clinical method, particularly in primary care, its empirical basis has been less well studied to date. Whereas needle acupuncture studies have shown activation and deactivation of the somatosensory cortex [3], [4], [6], [7], [11]–[14], superficial needling and laser intervention appear to stimulate cortical and subcortical structures other than the somatosensory cortex [11], [19], [20]. This is consistent with the observation that low intensity laser stimulation does not produce a skin sensation. For example, laser acupuncture of a foot acupoint, classically used for treating visual problems, was demonstrated to cause activation of the occipital cortex [19].

This study has used laser delivered at low intensity as used in primary care. Other studies have reported high intensity lasers can produce de qi sensation [21]. High intensity laser is not commonly used in primary care situations and therefore was not used in the current study. Further, as low intensity laser does not result in sensory sensation it is ideal for double blind randomized controlled studies where the subjects could not differentiate between placebo (laser off) and verum laser (laser on).

**Figure 1:**
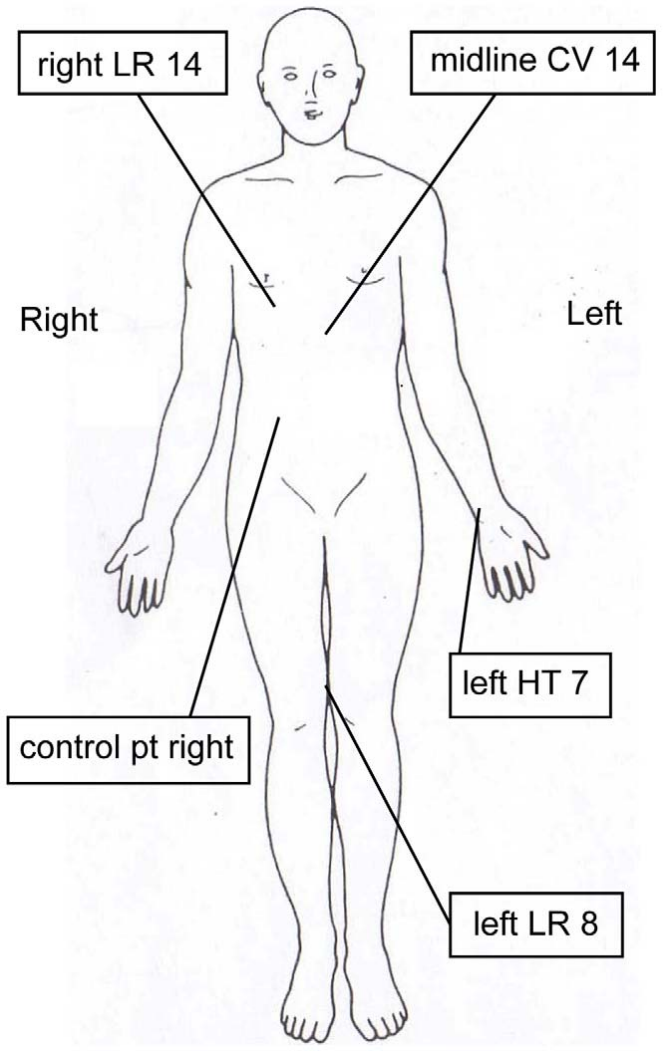
Selected acupoints relevant to mood and depression. In Traditional Chinese Medicine, the Liver (LR) meridian is used to treat depression. Two LR points were used: LR14 and LR 8. Two other acupoints considered important in depression were also used (CV 14 and HT 7). The control point (sham point or non acupoint) was located midway between SP15 and ST 25 acupoints.

The evidence suggests that cortical activation does occur with acupuncture and this activation may be specific to certain brain regions in relation to the site and type of stimulation [11]–[14]. In practice, acupoint efficacy is not specific, and one acupoint can be used for several different conditions, just as one medical condition can be managed with several acupoint locations. For instance, the antidepressant effect of laser acupuncture [22] has been attributed to a group of acupoints - CV14, LR14, LR8 and HT7 (see fig.1 for anatomical location), however there are other acupoint combinations that are also applicable for the management of depression. The neurological effects of stimulation of these acupoints CV14, LR14, HT7 and LR8 in combination have yet to be investigated.

In this study, we examined the blood oxygen level dependant (BOLD) functional magnetic resonance imaging (fMRI) response to laser simulation on the above mentioned acupoints CV14, LR14, LR8 and HT7. We chose laser acupuncture as it permits blinding of application because of the lack of a local sensation at low intensity, together with the previously mentioned increases in practical usage and limited understanding of its mechanisms. We reasoned that if laser acupuncture is altering a person's mental state a neurological effect should be observable. Further, if the effect differs dependent on the site of stimulation, then the neural locus of the activity should also differ.

## Materials and Methods

### Ethics Statement

The study was approved by the human research ethics committee of the South Eastern Sydney & Illawarra Area Health Service and participants provided written informed consent before participation.

### Participants

The participants (n = 10) (7 women, 3 men) were healthy volunteers aged 18–50 years (mean age  = 39.8 years) who were recruited by advertisement from the staff and students of the University of New South Wales and Prince of Wales Hospital, Sydney, Australia. All participants were right-handed and had no past history of depression or other psychiatric disorder, a Beck Depression Inventory [21] score <10, no history of drug or alcohol abuse, no medication intake within 3 months of the study, and no neurological or systemic disorders. Eight were acupuncture naïve and two had had needle acupuncture more than 3 months previously and did not know what to expect from laser intervention. Any contra-indications to MRI (pacemaker, ferromagnetic implants or foreign body, claustrophobia) were exclusionary.

### Choice of acupoints and control point

The acupoints were selected based on results from our previous study [22] and the TCM for mood disorders [23], [24]. These acupoints lay on the classically named liver (LR), heart (HT) and conception vessel (CV) meridians. The selected points, labelled LR8, LR14, HT7 and CV14 in TCM, are shown in Figure 1. LR8 is in the medial knee region, between the insertions of the sartorius and semitendinosus muscles. LR14 is in the vicinity of the 6th intercostal space on the mid clavicular line. HT7 is at the wrist crease, in the vicinity of the radial side of the flexor carpi ulnaris. CV14 is in the anterior midline, approximately 5 cm below the xiphisternum. A control non-acupoint was selected on the abdominal surface, midway between SP15 (four cun from the umbilicus) and ST25 (two cun from the umbilicus) away from the abdominal meridians.

### fMRI design

A block design was used, with each block of 20 seconds duration during which the subject received either laser stimulation (switched ‘on’) or placebo stimulation (switched ‘off’) at one acupoint. The infra red laser was held with light touch on the skin surface by the acupuncturist. Since the laser produces no sensation, the subject was able to be kept blind to the phase of stimulation. The on-off cycle was repeated 4 times for each acupoint (LR14, LR8, CV14, HT7), with the 4 acupoints being stimulated twice in random order. The control point near ST25 was stimulated once per subject. The block design accomodated for the placebo (laser off) condition during its rest phases. In total there were nine runs per subject. The subject was told to relax and keep his/her eyes closed during the entire time in the scanner.

### Laser stimulation

A Moxla^R^ prototype fiberoptic infra-red light laser (808 nm) with 25 mW capacity and a fiber optic arm was developed for usage in the scanning room. The laser parameters are similar to the one used in the clinical study we have based our investigation upon [22]. The acupoints were marked with a skin marking pencil prior to entry into the scanning room. A stably held laser was applied to the skin by the acupuncturist (IQ-S) who moved it from point to point according to the time signal. The switching on and off was achieved with a computer signal time-locked to the MRI acquisition.

### fMRI

Imaging was performed on a 3T Philips Intera MRI scanner (Philips Medical Systems, Best, Netherlands) for both T1-weighted 3D structural and BOLD contrast functional MRI. The 3D structural MRI was acquired in sagittal orientation using a T1-weighted TFE sequence (TR/TE  = 6.39/2.9 ms; flip angle  = 8; matrix size  = 256×256; FOV  = 256×256 mm; slice thickness 1.0 mm), yielding sagittal slices of 1.0 mm thick and an in-plane spatial resolution of 1.0×1.0 mm, producing an isotropic voxel of 1.0×1.0×1.0 mm. A gradient echo-planar imaging (EPI) technique (TR/TE = 2000/40 ms; matrix size  = 128×128; FOV = 250×250 mm; in plane pixel size 1.953×1.953 mm) was used to acquire T2-weighted BOLD contrast fMRI in axial orientation. The whole brain was covered using 21 slices at 5.0 mm slice thickness and 0.5 mm gap for each volume. Each session of 96 volumes were collected with the rate of 2s/volume.

### Image preprocessing and statistical analysis

Imaging data were analyzed using statistical parametric mapping (SPM2, Wellcome Department of Cognitive Neurology, London, UK) implemented in Matlab version 6 (The Mathworks Inc., USA). All volumes were realigned spatially to the first volume and the time-series for voxels within each slice realigned temporally to acquisition of the middle slice. Resulting volumes were normalized to a standard EPI template based on the Montreal Neurological Institute (MNI). The normalized images were smoothed with an isotropic 8 mm full-width half-maximal Gaussian kernel. The time-series in each voxel were highpass-filtered to 1/120 Hz to remove low-frequency noise.

Statistical analysis was performed in two stages, assuming a random effects design. Each stimulation site was compared to the placebo (laser off) condition for first level analysis. The BOLD response to the laser acupuncture stimulation was modeled by a canonical hemodynamic response function (HRF). The second level analysis (ANOVA) used each individual subject's contrast images, which were effectively the statistical parametric maps of the t-statistics for each voxel. The data had a threshold of p <0.001 with a spatial extent of 15 contiguous voxels.

### Post-imaging Assessment

After the scanning session, subjects rated selected items on the Spielberger State Anxiety Inventory [25] to describe their mental state during the period of the scanning. The ratings were: 1 (not at all), 2 (somewhat), 3 (moderately so) and 4 (very much so).

## Results

### Group analysis

At the group level, there were significant increases (activation) in BOLD levels in some brain regions for acupoints LR14, CV14, LR8 and the control point compared to all the other points (verum laser per point > all others, p<0.001; see Table 1). Further, there were significant decreases (deactivation) in BOLD levels for acupoints LR14, LR8 and the control point compared to all the other points (all others > verum laser per point, p<0.001; see Table 1) in other brain regions.

**Table 1:** Significant brain activation patterns from laser acupuncture to LR14, CV14, LR8 and control point.

ACUPOINT	x	y	z	p_vox_	No of Voxels	Brain Region
**Activation**						
LR14	−28	−42	68	0	169	Left Postcentral Gyrus
	−26	−6	62	0	85	Left Superior Frontal Gyrus
	−44	34	26	0	20	Left Middle Frontal Gyrus
CV14	−10	−50	6	0	84	Left Posterior Cingulate
LR8	−6	0	42	0	48	Left Cingulate Gyrus
Control Point	50	−26	48	0	40	Right Postcentral Gyrus
**Deactivation**						
LR14	−26	−42	−32	0	54	Left Cerebellar Tonsil
	−8	−66	18	0	69	Left Occipital Lobe, Precuneus
LR8	36	6	52	0	96	Right Middle Frontal Gyrus
	−26	12	62	0	83	Left Middle Frontal Gyrus
	20	14	54	0	151	Right Superior Frontal Gyrus
	42	−60	22	0	131	Right Middle Temporal Gyrus
	10	8	8	0	35	Right Caudate
Control Point	−26	−56	−4	0	182	Left Parahippocampal Gyrus

With LR8, activation of ipsilateral limbic cortex (cingulate gyrus) and deactivation of bilateral frontal cortices (middle frontal gyrus), (contralateral superior frontal gyrus), contralateral temporal cortex (middle temporal gyrus) and contralateral caudate occurred. Stimulation at the LR14 acupoint resulted in activation of contralateral frontal cortex (superior and middle frontal gyrii), contralateral parietal cortex (postcentral gyrus) and deactivation of contralateral cerebellum (cerebellar tonsil) and contralateral occipital cortex (precuneus). Acupoint CV14 produced activation of the left limbic cortex in the posterior cingulate and there was no significant deactivation in the grey matter. HT7 had no significant activation or deactivation. The control point (non acupoint or sham point), activated the contralateral parietal cortex (postcentral gyrus). It also deactivated the contralateral limbic cortex (parahippocampal gyrus).

### Somatosensory cortex and laterality of cerebral activation and deactivation

Our study involved randomized stimulation of the 4 acupoints and a control point. Although there was activation of contralateral postcentral gyrus (primary somatosensory cortex or SSI) with LR14 and control point, none of the other acupoints showed any activation of the somatosensory cortex. The cortical and subcortical structures activated with stimulation of the limb acupoints tended to be largely ipsilateral to the side of stimulation.

All the acupoints and control point did not have deactivation at the somatosensory cortex. However they all had contralateral deactivations with the exception of LR8 that had bilateral middle frontal gyrus deactivation.

### Behavioral observations

Participants did not describe anxiety or discomfort during scanning, except for one who found the headphones uncomfortable. The mean Spielberger scale ratings were on select items were: feeling calm (3.3), secure (3.3), relaxed (3.3), nervous (1.4), jittery (1.1), worried (1.1) or overexcited and rattled (1.1).

## Discussion

This is the first fMRI study to examine the effects of laser stimulation of a suite of acupoints found to be efficacious in a clinical condition (depression). A salient feature of this study was that four acupoints and a control non-acupoint (sham point) were stimulated in a random design. The subjects were unaware of the relative significance of different acupoints. The use of low level laser acupuncture, which does not produce a skin sensation, permitted the blinding of subjects to verum or placebo stimulation, something difficult to achieve with needle acupuncture.

The main finding of our study was that each acupoint or control point resulted in a different pattern of brain activity when contrasted against all the other acupoints or control point. The acupoints we investigated in this study were those that have been used in our previous treatment study for depression [22]. This finding suggests that although these acupoints are all used in the treatment of depression, the neural locus of this effect differs depending upon the site stimulated. The efficacy of these acupoints in the treatment of depression may vary greatly between patients and site stimulated, and our findings may shed some light on these effects [26].

The neuroanatomical basis of depression is not completely understood, however a number of studies have implicated abnormalities in certain brain regions, in particular the medial and dorsolateral prefrontal cortex, the cingulate gyrus and the so-called limbic brain regions (hippocampus, parahippocampal gyrus, amygdala, septal nuclei, insula, thalamus) and paralimbic regions (orbitofrontal cortex, anterior temporal lobe) [26]–[29]. There is converging evidence from drug treatment, cognitive-behavior therapy and brain stimulation techniques that antidepressant treatments work by modulating frontal-subcortical neuronal circuits. The most consistently reported finding is that antidepressant treatments lead to a normalization of activity in the dorsolateral prefrontal cortex, with additional changes in the subgenual cingulate region, the posterior cingulate, parahippocampal gyrus and insula [29]. Whether the change in prefrontal cortex is a primary event or secondary to changes in subcortical nuclei is unclear, but the relationship of treatment response to this suggests that it is biologically plausible that laser acupuncture could be an effective antidepressant treatment through its effects on the above brain regions.

The results show this combination of acupoints activating frontal cortex, limbic cortex and subcortical caudate. The trend is for ipsilateral activation suggestive of neurological circuitry outside the dorsal spinal columns and more likely to be autonomically driven [30]–[32]. Most of the deactivations were contralateral. Also LR14 and control point activations included primary somatosensory cortical activations (SSI). None of the deactivations involved SSI, however they did include the regions as described earlier that could collectively be called the affective cortex (the frontal, limbic and temporal cortices as well as the subcortical caudate). This combination of ipsilateral and contralateral activations and deactivations may perhaps be representative of the combined actions of both the spinal and autonomic nervous systems during laser acupuncture.

In classical acupuncture, there are primary and secondary acupoints for the treatment of any disorder. The approach to acupoint selection can be variable, with primary acupoints being considered essential and secondary acupoints additive for some patients. In our study, we cannot predict from these results whether any acupoint should be preferred over others for clinical use, even though LR8 deactivated more brain regions (middle and superior frontal gyrus, middle temporal gyrus and the subcortical caudate) than all the other points. These are results from a sample of healthy subjects. The question of whether the results would be different in a sample of clinically depressed subjects, needs to still be answered. Further studies are required to explore the relative value of different acupoints, the final test for which naturally lies in a clinical trial. It also cannot be stated from our study whether the treatment response can be achieved with stimulation at one point alone, or if multiple points are necessary.

There is conflicting evidence regarding acupoint specificity and whether that specificity is relative rather than absolute for any particular disorder [33]–[38]. Furthermore, it is debatable whether the clinical effects of acupuncture are restricted to stimulation on points that lie on the classical meridians in TCM. Our finding that laser stimulation of a non-acupoint produced some brain activation suggest that there is unlikely to be a completely neutral control non-acupoint, and this should prompt a re-examination of the use of sham points (in needle acupuncture studies) as control hence minimizing the true statistical effects of any acupoint [36]–[39]. It is also interesting that laser acupuncture in this study appeared to preferentially activate the limbic cortex ipsilaterally and deactivate the limbic cortex contralaterally. It has been suggested that laser stimulation preferentially activates unmyelinated afferent fibers that project ipsilaterally to the insula [40]–[42], which might also explain the differences from needle acupuncture.

This laser acupuncture fMRI study demonstrated the central effects of stimulation of a suite of acupoints found to be efficacious in treating depression in a primary care setting. The multiple acupoints each activated different groupings of frontal-limbic-striatal brain regions, suggesting some acupoint specificity but also a commonality in the regions affected. There was a trend for the limb acupoints to cause ipsilateral activation and contralateral de-activation. The results of the study suggest that laser acupuncture is a biologically plausible anti-depressant treatment. Its efficacy and the relative merits of the different proposed acupoints must be empirically examined.

## References

[1] Jorm AF, Christensen H, Griffiths KM, Rodgers B (2002). Effectiveness of complementary and self-help treatments for depression.. Med J Aust.

[2] Kessler RC, Soukup J, Davis RB, Foster DF, Wilkey SA (2001). The use of complementary and alternative therapies to treat anxiety and depression in the United States.. Am J Psychiatry.

[3] Hui KK, Liu J, Marina O, Napadow V, Haselgrove C (2005). The integrated response of the human cerebro-cerebellar and limbic systems to acupuncture stimulation at ST 36 as evidenced by fMRI.. NeuroImage.

[4] Napadow V, Dhond RP, Purdon P, Kettner N, Makris N (2005). Correlating acupuncture FMRI in the human brainstem with heart rate variability.. Conf Proc IEEE Eng Med Biol Soc.

[5] Napadow V, Makris N, Liu J, Kettner NW, Kwong KK (2005). Effects of electroacupuncture versus manual acupuncture on the human brain as measured by fMRI.. Hum Brain Mapp.

[6] Napadow V, Kettner NW, Liu J, Li M, Kwong KK (2007). Hypothalamus and amygdala response to acupuncture stimuli in carpal tunnel syndrome.. Pain.

[7] Hsieh JC, Tu CH, Chen FP, Chen MC, Yeh TC (2001). Activation of the hypothalamus characterises the acupuncture stimulation at the analgesic point in the human: a PET Study.. Neurosci Lett.

[8] Yoo SS, The EK, Blinder RA, Jolesz FA (2004). Modulation of cerebellar activities by acupuncture stimulation: evidence from fMRI study.. NeuroImage.

[9] Kong J, Gollub RL, Webb JM, Kong JT, Vangel MG (2007). Test-retest study of fMRI signal change evoked by electroacupuncture stimulation.. NeuroImage.

[10] Haker E, Egevist H, Bjerring P (2000). The effect of sensory stimulation (acupuncture) on symp and parasymp activities in healthy subjects.. J Auton Nerv Syst.

[11] Wu MT, Hsieh JC, Xiong J, Yang CF, Pan HB (1999). Central nervous system pathway for acupuncture stimulation: localisation of processing with fMRI of the brain – preliminary experience.. Radiology.

[12] Fang JL, Krings T, Weidemann J, Meister IG, Thron A (2004). Functional MRI in healthy subjects during acupuncture: different effects of needle rotation in real and false acupoints.. Neuroradiology.

[13] Zhang WT, Jin Z, Cui GH, Zhang KL, Zhang L (2003). Relations between brain network activation and analgesic effect induced by low vs. high frequency electrical acupoint stimulation in different subjects: a functional magnetic resonance imaging study.. Brain Res.

[14] Geng Li, Clifford RJ, Yang ES (2006). An fMRI study of somatosensory implicated acupuncture points in stable somatosensory stroke patients.. J Magn Reson Imaging.

[15] Qin W, Bai L, Yang L, Chen P, Dai J (2008). FMRI connectivity analysis of acupuncture effect on an amygdala-associated brain network.. Mol Pain.

[16] Dhond RP, Yeh C, Park KY, Kettner N, Napadow V (2008). Acupuncture modulates resting state connectivity in default and sensorimotor brain networks.. Pain.

[17] Rogers BP, Morgan VL, Newton AT, Gore JC (2007). Assessing functional connectivity in the human brain by fMRI.. Magnetic Res Imaging.

[18] Pariente J, White P, Frackowiak SJ, Lewith G (2005). Expectancy and belief modulate the neuronal substrates of pain treated by acupuncture.. NeuroImage.

[19] Siedentopf CM, Golaszewski SM, Mottaghy FM, Ruff CC, Felber S (2002). Functional magnetic resonance imaging detects activation of visual association cortex during laser acupuncture of the foot in humans.. Neurosci Lett.

[20] Siedentopf CM, Koppelstaetter F, Haala IA, Haid V, Rhomberg P (2005). Laser acupuncture induced specific cerebral cortical and subcortical activations in humans.. Lasers Med Sci.

[21] Rindge D (2009). http://www.acupuncturetoday.com/mpacms/at/article.php?id=31944.

[22] Quah-Smith JI, Tang WM, Russell J (2005). Laser acupuncture for mild to moderate depression in a primary care setting - a randomised controlled trial.. Acupunct Med.

[23] Maciocia G (1994). The practice of chinese medicine: The treatment of diseases with acupuncture and chinese herbs.. Churchill-Livingstone.

[24] Aung SKH, Chen WPD (2007). Clinical introduction to medical acupuncture..

[25] Spielberger CD, Gorsuch RL, Lushene R (1970). Manual for the state-trait anxiety inventory (self evaluation questionnaire): manual..

[26] Sheline YI (2003). Neuroimaging studies of mood disorder effects on the brain.. Biol Psychiatry.

[27] Reiman EM (1997). The application of positron emission tomography to the study of normal and pathologic emotions.. J Clin Psychiatry.

[28] George MS, Ketter TA, Post RM, Panksepp J, Greenwich CT (1996). What functional imaging studies have revealed about the brain basis of mood and emotion.. Advances in Biology Psychiatry.

[29] Goldapple K, Segal Z, Garson C, Lau M, Bieling P (2004). Modulation of cortical-limbic pathways in major depression: treatment-specific effects of cognitive behavior therapy.. Arch Gen Psychiatry.

[30] Wu JH, Chen HY, Chang YJ, Wu HC, Chang WD (2009). Study of autonomic nervous activity of night shift workers treated with laser acupuncture.. Photomed Laser Surg.

[31] Agelink MW, Sanner D, Eich H, Pach J, Bertling R (2003). Does acupuncture influence the cardiac autonomic nervous system in patients with minor depression or anxiety disorders?. Fortschr Neurol Psychiatr.

[32] Chang S, Chao WL, Chiang MJ, Li SJ, Lu YT (2008). Effects of acupuncture at Neiguan (PC6) of the pericardial meridian on blood pressure and heart rate variability.. Chin J Physiol.

[33] Campbell A (2006). Point specificity of acupuncture in the light of recent clinical and imaging studies.. Acupunct Med.

[34] Yan B, Li K, Xu JY, Wang W, Li KC (2005). Acupoint-specific fMRI patterns in human brain.. Neurosci Lett.

[35] Jin Z, Luo F, Zhang WT, Zhang L, Zeng YW (2004). Evidence from brain imaging with fMRI supporting functional specificity of acupoints in humans.. Neurosci Lett.

[36] Paterson C, Zheng Z, Xue C, Wang Y (2008). “Playing their parts”: The experiences of participants in a randomized sham controlled acupuncture trial.. J Altern Complement Med.

[37] Tsukayama H, Yamashita H, Kimura T, Otsuki K (2006). Factors that influence the applicability of sham needle in acupuncture trials.. Clin J Pain.

[38] Lundeberg T, Lund I (2009). Treatment recommendations should take account of individual patient variation not just group responses.. Acupunct Med.

[39] Lund I, Naslund J, Lundeberg T (2009). Minimal acupuncture is not a valid placebo control in randomized controlled trials of acupuncture: a physiologist's perspective. Chinese Medicine 4:1.. http://www.cmjournal.org/content/4/1/1.

[40] Paterson C, Dieppe OP (2005). Characteristic and incidental (placebo) effects in complex interventions such as acupuncture.. BMJ.

[41] Macpherson H, Green G, Nevado A, Lythgoe M, Lewith G (2008). Brain imaging of acupuncture: comparing superficial with deep needling.. Neurosci Lett.

[42] Olausson H, Lamarre Y, Backlund H, Morin C, Wallin BG (2002). Unmyelinated tactile afferents signal touch and project to the insular cortex.. Nat Neurosc.

